# Anxiety and depression prior to total knee arthroplasty are associated with worse pain and subjective function: A prospective comparative study

**DOI:** 10.1002/ksa.12336

**Published:** 2024-06-29

**Authors:** Margot B. Aalders, Jelle P. van der List, Lucien C. M. Keijser, Joyce L. Benner

**Affiliations:** ^1^ Centre for Orthopaedic Research Alkmaar (CORAL) Alkmaar The Netherlands; ^2^ Department of Orthopaedic Surgery NorthWest Clinics Alkmaar The Netherlands; ^3^ Department of Orthopaedic Surgery and Sports Medicine Amsterdam UMC location AMC Amsterdam The Netherlands; ^4^ Department of Orthopaedic Surgery Flinders University Adelaide South Australia Australia; ^5^ Amsterdam Movement Sciences Sports, Amsterdam The Netherlands; ^6^ Department of Human Movement Sciences, Faculty of Behavioral and Movement Sciences, Vrije Universiteit Amsterdam Amsterdam Movement Sciences Amsterdam The Netherlands

**Keywords:** anxiety, depression, Hospital Anxiety and Depression Scale, knee replacement, psychological factors, total knee arthroplasty

## Abstract

**Purpose:**

The aim of this study was to investigate the influence of preoperative anxiety and depression on subjective function, pain and revision rates following total knee arthroplasty (TKA).

**Methods:**

A prospective comparative study was conducted, including 349 patients undergoing TKA surgery between January 2019 and April 2021. Patients completed the Hospital Anxiety and Depression Scale (HADS) questionnaire preoperatively, and a set of Patient‐Reported Outcome Measures (PROMs) preoperatively and at 6, 12 and 24 months postoperatively. Patients were categorized into anxiety and depression groups based on HADS scores. PROMs included the Knee injury and Osteoarthritis Outcome Score‐Physical Function Shortform (KOOS‐PS), Oxford Knee Score (OKS) and NRS‐Pain. Differences in PROM scores between the anxiety/depression group and, respectively, nonanxiety/nondepression group were assessed, as well as differences in minimal clinical important difference (MCID) and attainment of Patient Acceptable Symptom State (PASS). Lastly, revision rates were compared.

**Results:**

Anxiety and depression groups exhibited inferior subjective function preoperatively and postoperatively compared to nonanxiety and nondepression groups (all *p* < 0.05), experienced more pain preoperatively (*p* < 0.001) and also postoperatively for depression patients (all *p* < 0.05). Significantly fewer patients with anxiety and depression reached the PASS for KOOS‐PS, OKS and NRS‐Pain (all *p* < 0.05). There were no differences in the proportion of patients reaching the MCID for all PROMs (all *p* > 0.060), and revision rates did not differ between groups (both *p* > 0.96).

**Conclusion:**

Preoperative anxiety and depression negatively influence subjective function and pain preoperatively and up to 2‐year follow‐up in patients undergoing TKA. Revision rates did not differ between groups, and there were no relevant differences in clinical improvement of subjective function and pain.

**Level of Evidence:**

Level II, prospective comparative study.

**Abbreviations**
BMI: body mass index
EQ‐5D: EuroQol five‐dimension
HADS: Hospital Anxiety and Depression Scale
HADS‐A: anxiety subscale of the HADS questionnaire
HADS‐D: depression subscale of the HADS questionnaire
KOOS‐PS: Knee injury and Osteoarthritis Outcome Score‐Physical Function Shortform
LOS: length of stay
MCID: minimal clinical important difference
NRS‐Pain: Numeric Rating Scale for pain
OA: osteoarthritis
OKS: Oxford Knee Score
PASS: Patient Acceptable Symptom State
PROMs: Patient‐Reported Outcome Measures
RECORD: Reporting of studies Conducted using Observational Routinely‐collected health Data
SD: standard deviation
STROBE: Strengthening the Reporting of Observational Studies in Epidemiology
TJA: total joint arthroplasty
TKA: total knee arthroplasty

## INTRODUCTION

Total knee arthroplasty (TKA) has grown to be the most common treatment for end‐stage osteoarthritis (OA) [44] with improvement of subjective function and pain postoperatively and a successful survival rate at long‐term follow‐up (range of 87%–99%) [15, 24, 35]. However, approximately 15% of patients appear to be dissatisfied postoperatively due to persisting or recurring pain symptoms and malfunction [4, 9, 37], despite a well‐functioning prosthesis clinically and radiographically [26]. Therefore, the most important predicting factors of dissatisfactory TKA outcomes should be elucidated.

Until now, the established associations determining postoperative dissatisfaction following TKA vary and remain unclear in the literature [7, 10, 16, 26, 29, 33, 37]. One of the potential factors include preoperative psychological factors, such as anxiety and depression. A few systematic reviews showed psychological factors are important predictors [33, 41, 45]. However, studies lack the assessment of anxiety and depression and have contradictory conclusions [33, 34, 38]. Furthermore, most of these studies are limited by small cohorts, a follow‐up of maximum 12 months, retrospective study designs or had no assessment of revision rate as an outcome measure [4, 34, 38, 47].

Therefore, the aim of this study was to fill this research gap and prospectively assess the role of preoperative anxiety and depression on subjective function, pain and revision rates after TKA within a larger cohort and a minimum 2‐year follow‐up. It was hypothesized that patients with preoperative anxiety or depression have inferior subjective function, more pain and higher revision rates following TKA.

## METHODS

### Study design

This concerned a prospective cohort study in a high‐volume teaching hospital in the Netherlands between 1 January 2019 and 30 April 2021 and was performed in accordance with the Declaration of Helsinki [48]. The institutional review board approved the study and written informed consents were obtained from all patients. The REporting of studies Conducted using Observational Routinely collected health Data (RECORD) and the Strengthening the Reporting of Observational Studies in Epidemiology (STROBE) guidelines were followed for data collection and reporting [2, 14].

### Participants

Patients were eligible for inclusion in this study if they (1) underwent unilateral primary TKA for symptomatic advanced OA [25], (2) spoke the Dutch language for completing questionnaires, (3) completed all preoperative questionnaires and (4) provided their informed consent. Patients were excluded if (1) follow‐up was less than 2 years, (2) TKA indication other than OA or (3) underwent TKA for revision surgery. To assess preoperative mental state, all patients completed a Patient‐Reported Outcome Measure (PROM): the Hospital Anxiety and Depression Scale (HADS) questionnaire [50], which consists of an anxiety (HADS‐A) and a depression (HADS‐D) subscale, each including seven items scoring 0–3, resulting in a total maximum score of 21 per subscale. Scores of 8–11 are considered borderline anxiety or depression, whereas scores >11 are considered presumable anxiety or depression [1]. In this study, a HADS‐A or HADS‐D score ≥8 was categorized as the presence of, respectively, anxiety or depression [6, 50]. Consequently, all patients were divided into an anxiety or nonanxiety group and into a depression or nondepression group. None of the patients received therapy for anxiety or depression symptoms within our institution.

**Figure 1:**
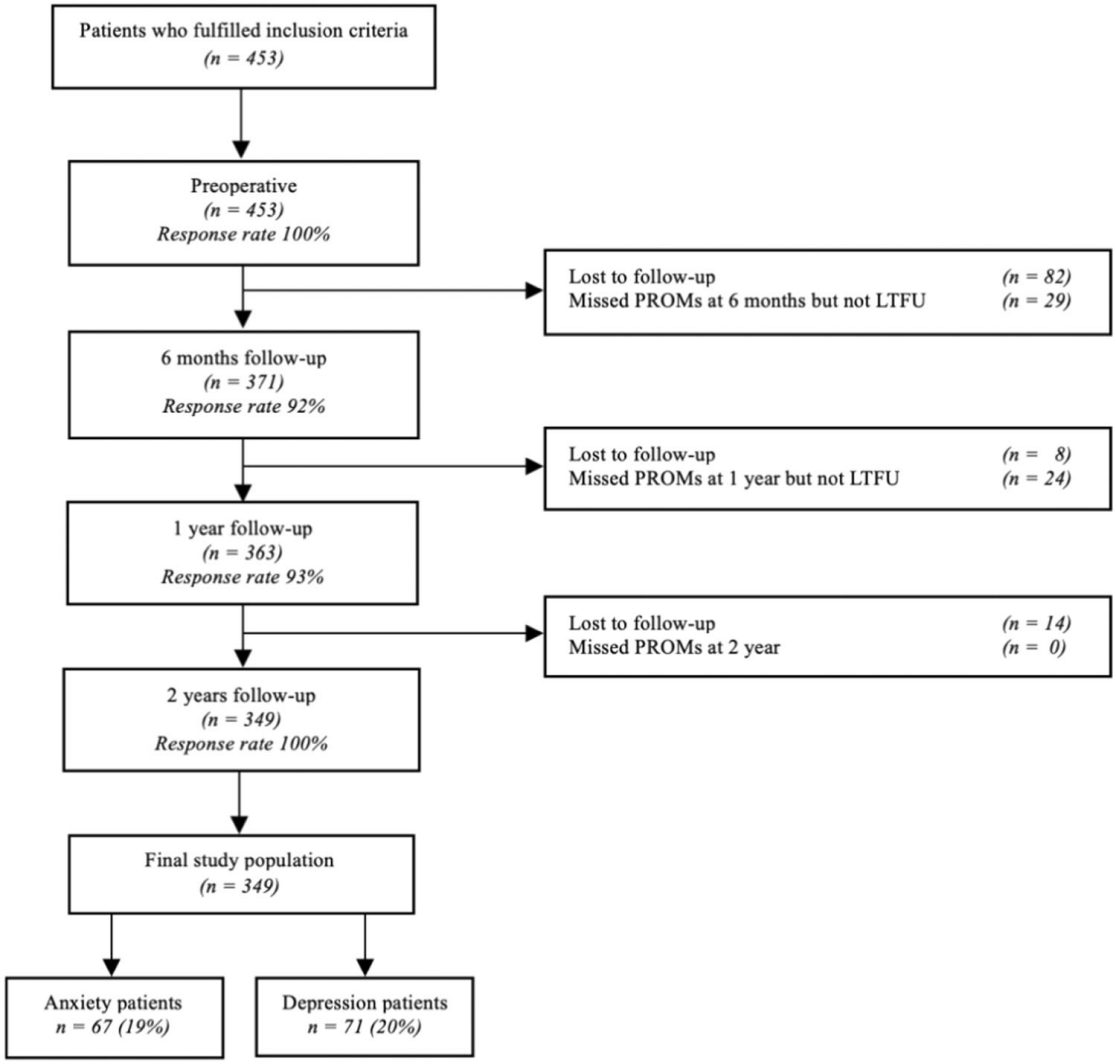
Flow diagram of the study population: patient selection and lost to follow‐up, including the response rate and number of drop‐outs, that is, excluded patients during the inclusion period. LTFU, lost to follow‐up; PROM, Patient‐Reported Outcome Measure. Microsoft Word was used to create this figure.

Within the specified timeframe, a total of 453 patients fulfilled all inclusion criteria. Of these, 104 were excluded for insufficient follow‐up (82 dropouts at 6 months, 8 dropouts at 1 year, 14 dropouts at 2 years) leading to a final cohort of 349 patients. A detailed flowchart of patient selection and loss to follow‐up is provided in Figure 1. A summary of patient, intra‐ and postoperative characteristics for all patients, and for patients stratified by anxiety and depression, can be found in Table 1. Preoperative anxiety and depression were present in, respectively, 67 patients (19%) and 71 patients (20%). No significant differences in characteristics were found between the anxiety versus nonanxiety groups, nor between the depression versus nondepression groups, except for length of stay (LOS) (days), which was longer in the anxiety and depression groups, and estimated blood loss (mL), which was higher in the anxiety group (Table 1).

**Table 1:** Baseline patient, intra‐ and postoperative characteristics for all patients and for patients stratified by anxiety and depression.

	All patients (*n* = 349)	Anxiety (*n* = 67)	Nonanxiety (*n* = 282)	*p* Value	Depression (*n* = 71)	Nondepression (*n* = 278)	*p* Value
Age (years)^a^	70 (63.0–74.0)	68 (61.0–74.0)	70 (63.0–74.3)	0.212	70 (63.0–76.0)	70 (63.0–74.0)	0.688
Female gender^b^	214 (61.3%)	46 (68.7%)	168 (59.6%)	0.170	45 (63.4%)	169 (60.8%)	0.689
BMI (kg/m^2^)^c^	29.3 ± 4.9	29.5 ± 5.3	29.3 ± 4.9	0.815	29.4 ± 5.5	29.3 ± 4.8	0.963
Smoking^b^	39 (11.2%)	10 (14.9%)	29 (10.3%)	0.278	10 (14.1%)	29 (10.4%)	0.383
ASA I/II^b^	253 (72.5%)	47 (70.1%)	206 (73%)	0.633	48 (67.6%)	205 (73.7%)	0.301
Mean HADS‐anxiety score^a^	4 (2.0–7.0)	11 (9.0–13.0)	3 (2.0–5.0)	**<0.001**	‐	‐	‐
Mean HADS‐depression score^a^	4 (2.0–6.0)	‐	‐	‐	9 (8.0–11.0)	3 (1.0–4.0)	**<0.001**
Medial parapatellar approach^b^	317 (91.1%)	61 (91%)	256 (91.1%)	0.773	65 (91.5%)	252 (91%)	0.773
Patellar resurfacing^b^	87 (24.9%)	14 (20.9%)	73 (25.9%)	0.396	17 (23.9%)	70 (25.2%)	0.830
Surgery duration (mins)^a^	70 (61.0–82.0)	74 (61.0–90.0)	70 (61.0–80.0)	0.060	70 (59.0–85.0)	70 (61.0–81.0)	0.471
Estimated blood loss (mL)^a^	150 (0.0–250.0)	200 (50.0–300.0)	100 (0.0–225.0)	**0.010**	150 (0.0–300.0)	150 (0.0–250.0)	0.177
Length of stay (days)^c^	2.4 ± 1.6	2.9 ± 2.0	2.2 ± 1.5	**0.013**	2.8 ± 1.9	2.3 ± 1.5	**0.041**
All‐cause revision (<2 years)^b^	8 (2.3%)	1 (1.5%)	7 (2.5%)	0.627	2 (2.8%)	6 (2.2%)	0.741
Aseptic revisions^b^	5 (1.4%)	1 (1.5%)	4 (1.4%)	0.963	1 (1.4%)	4 (1.4%)	0.985
Arthrofibrosis^b^	2 (0.5%)	1 (1.5%)	1 (0.3%)		1 (1.4%)	1 (0.3%)	
Patella addition^b^	3 (0.9%)		3 (1.1%)			3 (1.1%)	

*Note*: Bold values indicate statistically significant.

Abbreviations: ASA, American Society of Anaesthesiologists physical status classification; BMI, body mass index; n, number of patients.

^a^ Reported in median with interquartile range (lower quartile–upper quartile).

^b^ Reported in number (percentage).

^c^ Reported in mean ± standard deviation.

### Surgical procedure

All patients received a posterior‐stabilized Genesis II with cemented tibial and femoral components and a standard polyethylene insert (Smith & Nephew). A patellar component was placed upon the surgeons indication. Surgeries were performed by a group of five orthopaedic surgeons, of which four used the medial parapatellar approach and one the subvastus approach. All patients received protocolized aftercare, which consisted of a 4‐week antithrombotic prophylaxis (heparin), mobilization with physical therapy and 1–3 weeks 50% weight bearing, gradually building up to 100%. Crutches were used during at least the first 2 weeks postoperatively. Outpatient check‐up with the surgeon took place at 8 weeks and 12 months postoperatively with radiographs.

### Data collection and outcomes

Baseline patient demographics (age, gender, body mass index, smoking, American Society of Anaesthesiologists physical status classification physiological status classification, side) and intra‐ and postoperative characteristics (surgical approach, patellar resurfacing, surgery duration, estimated blood loss, length of hospital stay) were collected through the electronic health‐record system. Aseptic revision rates and failures were first checked in the health‐record system, and additionally, the Dutch Arthroplasty Registry was consulted for any revisions performed outside our institution.

Primary outcome measures were the PROMs for subjective function and pain. Patients were invited to complete a set of PROMs before surgery at 6, 12 and 24 months postoperatively, for which two automatic reminders were mailed. In case of nonresponse despite the reminders, patients were considered lost to follow‐up. PROMs included the Knee injury and Osteoarthritis Outcome Score‐Physical Function Shortform (KOOS‐PS), Oxford Knee Score (OKS) and Numeric Rating Scale for pain (NRS‐Pain). Additionally, clinical improvements (Δ) between the preoperative score and follow‐up moments were established for each PROM, and frequencies of patients with delta scores exceeding the minimal clinical important difference (MCID) were determined. Lastly, frequencies of patients reaching the Patient Acceptable Symptom State (PASS) were established for each PROM at all follow‐up moments. For the KOOS‐PS, a MCID of 10 and a PASS of 63.7 were used [3, 27] for OKS 5 and 37 [8, 22] and for NRS‐Pain 18 and 25 [9, 32].

A secondary outcome measure included aseptic revision surgery. We included aseptic revision surgery as an outcome measure due to the premise that persistent prosthetic pain lowers the threshold for revision surgery. Thus, even minor malpositioning (not necessarily the primary cause of discomfort) may prompt revision in these patients. Conversely, infection represents a distinct failure scenario with objective criteria less influenced by pain presentation. Therefore, we opted not to include septic revision surgery as an outcome measure.

### Statistical Analysis

Baseline patient demographics, intra‐ and postoperative characteristics and PROMs scores were described as the mean with standard deviation (SD) for continuous variables and as number with percentage (%) for categorical variables. When normality assumptions were violated, the median (mn) and interquartile range (IQR) were presented. Unpaired *t*‐tests were used to compare all PROMs (improvement) scores between groups (nonanxiety vs. anxiety, nondepression vs. depression). Similarly, *χ* ^2^ tests were used to compare categorical variables between groups and Mann–Whitney *U*‐tests for nonnormal distributed variables.

The sample size calculation of the KOOS‐PS using an SD of 17.3 points (preliminary data), *α* 0.05, power 80% and enrolment ratio 4:1 revealed 29 patients with depression or anxiety and 150 nondepression or nonanxiety patients were needed to calculate a MCID of 10 points in the KOOS‐PS. Similarly for OKS, sample size calculation was performed with a SD of 8.9 points, showing that 31 patients with depression or anxiety and 124 patients who are nondepression or nonanxiety patients were needed to calculate a MCID of 5 points [21]. All data were analysed using SPSS (version 28.0; IBM Corp.) and a *p* value of 0.05 was considered statistically significant. Sources of bias were addressed by the prospective study design, a large study population with a follow‐up of at least 2 years and by limiting the number of lost‐to‐follow‐up patients through sending reminder emails.

## RESULTS

**Figure 2:**
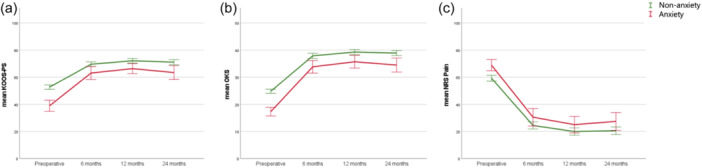
Anxiety and nonanxiety patients: Course of the subjective function (a: Knee injury and Osteoarthritis Outcome Score‐Physical Function Shortform and b: Oxford Knee Score) and joint pain (c: Numeric Rating Scale‐pain), from preoperative to all follow‐up moments, for anxiety (in red) and nonanxiety (in green) patients. Corresponding Tables 2, 3, 4. SPSS was used to create this figure.

**Figure 3:**
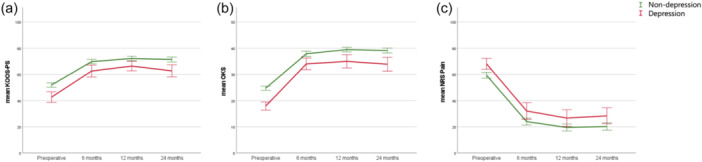
Depression and nondepression patients: Course of the subjective function (a: Knee injury and Osteoarthritis Outcome Score‐Physical Function Shortform and b: Oxford Knee Score) and joint pain (c: Numeric Rating Scale‐pain), from preoperative to all follow‐up moments, for depression (in red) and nondepression (in green) patients. Corresponding Tables 2, 3, 4. SPSS was used to create this figure.

Both anxiety as well as depression patients had significantly inferior subjective function preoperatively (for KOOS‐PS as well as OKS, all *p* < 0.001) and at all postoperative follow‐up moments (all *p* < 0.05), compared to the nonanxiety and nondepression patients, respectively (Figures 2 and 3 and Tables 2 and 3). Similar results were observed when correcting for baseline patient characteristics (in all multivariate analyses all *p* < 0.011).

**Table 2:** KOOS‐PS scores for all patients and for patients stratified by anxiety and depression.

	All patients (*n* = 349)	Anxiety (*n* = 67)	Nonanxiety (*n* = 282)	*p* Value	Depression (*n* = 71)	Nondepression (*n* = 278)	*p* Value
KOOS‐PS preoperative^a^	50.0 ± 15.3	38.9 ± 17.1	52.7 ± 13.6	**<0.001**	42.7 ± 17.0	51.9 ± 14.3	**<0.001**
KOOS‐PS 6 months^a^	68.3 ± 15.2	63.1 ± 19.1	69.6 ± 13.9	**0.013**	62.6 ± 18.4	69.7 ± 14.0	**<0.001**
KOOS‐PS 12 months^a^	71.0 ± 14.5	66.3 ± 14.6	72.1 ± 14.3	**0.004**	66.4 ± 14.7	72.1 ± 14.3	**0.004**
KOOS‐PS 24 months^a^	69.6 ± 17.1	63.4 ± 20.8	71.1 ± 15.8	**0.006**	62.7 ± 19.5	71.4 ± 16.0	**<0.001**
KOOS‐PS Δ 0–6 months^a^	18.3 ± 17.8	24.7 ± 23.9	16.7 ± 15.6	**0.014**	20.4 ± 22.9	17.8 ± 16.3	0.379
KOOS‐PS Δ 0–12 months^a^	21.0 ± 17.3	27.1 ± 17.5	19.5 ± 16.9	**0.002**	24.2 ± 19.2	20.2 ± 16.7	0.097
KOOS‐PS Δ 0–24 months^a^	19.6 ± 18.6	24.5 ± 21.4	18.4 ± 17.7	**0.033**	20.0 ± 21.5	19.4 ± 17.8	0.840
MCID Δ 0–6 months^b^	222 (68.9%)	47 (74.6%)	175 (67.6%)	0.279	47 (72.3%)	175 (68.1%)	0.512
MCID Δ 0‐12 months^b^	244 (74.8%)	53 (84.1%)	191 (72.6%)	0.059	51 (78.5%)	193 (73.9%)	0.453
MCID Δ 0–24 months^b^	246 (70.5%)	51 (76.1%)	195 (69.1%)	0.261	48 (67.6%)	198 (71.2%)	0.551
PASS 6 months^b^	220 (68.3%)	38 (60.3%)	182 (70.3%)	0.128	39 (60.0%)	181 (70.4%)	0.106
PASS 12 months^b^	228 (69.9%)	34 (54.0%)	194 (73.8%)	**0.002**	36 (55.4%)	192 (73.6%)	**0.004**
PASS 24 months^b^	231 (66.2%)	36 (53.7%)	195 (69.1%)	**0.016**	38 (53.5%)	193 (69.4%)	**0.011**

*Note*: Twenty‐nine patients missed outcomes at 6 months, twenty‐four patients missed outcomes at 12 months and nine patients missed outcomes at both 6 and 12 months. All patients had outcomes preoperatively and at 24 months. Bold values indicate statistically significant.

Abbreviations: KOOS‐PS, Knee injury and Osteoarthritis Outcome Score‐Physical function Shortform; MCID, minimal clinical important difference; n, number of patients; PASS, patient acceptable symptom state.

^a^ Reported in mean ± standard deviation.

^b^ Reported in number (percentage).

**Table 3:** Oxford Knee Score (OKS) scores for all patients and for patients stratified by anxiety and depression.

	All patients (*n* = 349)	Anxiety (*n* = 67)	Nonanxiety (*n* = 282)	*p* Value	Depression (*n* = 71)	Nondepression (*n* = 278)	*p* Value
OKS preoperative^a^	23.4 ± 7.2	17.3 ± 6.5	24.8 ± 6.6	**<0.001**	17.9 ± 6.5	24.7 ± 6.7	**<0.001**
OKS 6 months^a^	37.1 ± 8.3	33.8 ± 9.2	37.9 ± 7.9	**0.002**	34.0 ± 9.0	37.9 ± 7.9	**0.002**
OKS 12 months^a^	38.6 ± 8.2	35.7 ± 9.2	39.3 ± 7.7	**0.002**	35.0 ± 10.2	39.5 ± 7.3	**<0.001**
OKS 24 months^a^	38.0 ± 8.9	34.5 ± 10.6	38.9 ± 8.2	**0.002**	33.9 ± 11.0	39.1 ± 7.9	**<0.001**
OKS Δ 0–6 months^a^	13.7 ± 8.5	16.7 ± 8.8	13.0 ± 8.3	**0.002**	16.6 ± 9.0	13.0 ± 8.3	**0.002**
OKS Δ 0–12 months^a^	15.1 ± 8.5	18.3 ± 9.1	14.3 ± 8.2	**<0.001**	17.4 ± 11.0	14.5 ± 7.7	0.053
OKS Δ 0–24 months^a^	14.7 ± 8.9	17.2 ± 9.8	14.1 ± 8.6	**0.010**	16.0 ± 11.1	14.4 ± 8.3	0.260
MCID Δ 0–6 months^b^	276 (86.0%)	57 (90.5%)	219 (84.9%)	0.252	60 (92.3%)	216 (84.4%)	0.100
MCID Δ 0–12 months^b^	290 (89.0%)	59 (93.7%)	231 (87.8%)	0.186	56 (86.2%)	234 (89.7%)	0.420
MCID Δ 0–24 months^b^	306 (87.7%)	60 (89.6%)	246 (87.2%)	0.604	60 (84.5%)	246 (88.5%)	0.362
PASS 6 months^b^	199 (62.0%)	31 (49.2%)	168 (65.1%)	**0.020**	31 (47.7%)	168 (65.6%)	**0.008**
PASS 12 months^b^	224 (68.7%)	37 (58.7%)	187 (71.1%)	0.057	37 (56.9%)	187 (71.6%)	**0.022**
PASS 24 months^b^	231 (66.2%)	34 (50.7%)	197 (69.9%)	**0.003**	36 (50.7%)	195 (70.1%)	**0.002**

*Note*: Twenty‐nine patients missed outcomes at 6 months, twenty‐four patients missed outcomes at 12 months and nine patients missed outcomes at both 6 and 12 months. All patients had outcomes preoperatively and at 24 months. Bold values indicate statistically significant.

Abbreviations: MCID, minimal clinical important difference; n, number of patients; PASS, patient acceptable symptom state; OKS, Oxford Knee Score.

^a^ Reported in mean ± standard deviation.

^b^ Reported in number (percentage).

Anxiety patients had significantly more clinical improvement in subjective function (KOOS‐PS and OKS) for all time intervals compared to nonanxiety patients (all *p* < 0.05). Depression patients had comparable clinical improvement in subjective function (KOOS‐PS and OKS) compared to nondepression patients, except on the ∆ 0–6 months interval for the OKS, in which depression patients showed more improvement (*p* = 0.002). However, there was no difference for both the anxiety and depression groups, compared to nonanxiety and nondepression groups, in the proportion of patients reaching the MCID. Significantly fewer patients with anxiety and depression compared to nonanxiety and nondepression patients reached the PASS for the KOOS‐PS and OKS (Tables 2 and 3).

Anxiety patients showed significantly more pain preoperatively compared to nonanxiety patients (*p* < 0.001), but not during follow‐up (Figure 2c and Table 4). Similar results were observed when correcting for baseline patient characteristics (in multivariate analysis *p* < 0.001). Depression patients had significantly more pain preoperatively (*p* < 0.001), and at all postoperative follow‐up moments (all *p* < 0.05) compared to the nondepression patients (Figure 3c and Table 4). Similar results were observed when correcting for baseline patient characteristics (in all multivariate analyses all *p* < 0.043). Clinical improvement was comparable between the anxiety and nonanxiety group and depression and nondepression group, as were the proportions of patients reaching the MCID of the NRS‐Pain at all time intervals. However, significantly fewer patients with anxiety and depression compared to nonanxiety and nondepression patients reached the PASS for NRS‐Pain at 24‐months and 12‐months follow‐up, respectively (Table 4).

**Table 4:** Numeric Rating Scale for pain (NRS‐Pain) scores for all patients and for patients stratified by anxiety and depression.

	All patients (*n* = 349)	Anxiety (*n* = 67)	Nonanxiety (*n* = 282)	*p* Value	Depression (*n* = 71)	Nondepression (*n* = 278)	*p* Value
NRS‐Pain preoperative^a^	61.1 ± 18.7	68.9 ± 16.9	59.2 ± 18.7	**<0.001**	68.0 ± 17.7	59.3 ± 18.6	**<0.001**
NRS‐Pain 6 months^a^	25.6 ± 22.4	30.5 ± 25.7	24.4 ± 21.5	0.086	32.1 ± 25.8	24.0 ± 21.2	**0.021**
NRS‐Pain 12 months^a^	20.9 ± 22.7	25.0 ± 24.3	19.9 ± 22.3	0.111	26.7 ± 25.6	19.5 ± 21.8	**0.039**
NRS‐Pain 24 months^a^	21.9 ± 24.2	27.4 ± 26.7	20.6 ± 23.4	0.058	28.4 ± 26.0	20.2 ± 23.5	**0.011**
NRS‐Pain Δ 0–6 months^a^	−35.7 ± 25.3	−38.4 ± 26.0	−35.0 ± 25.1	0.340	−35.7 ± 26.7	−35.7 ± 24.9	0.997
NRS‐Pain Δ 0–12 months^a^	‒40.2 ± 27.3	−43.4 ± 25.4	−39.4 ± 27.7	0.294	−41.8 ± 29.5	−39.8 ± 26.7	0.580
NRS‐Pain Δ 0–24 months^a^	−39.2 ± 27.6	−41.5 ± 27.3	−38.7 ± 27.7	0.453	−39.6 ± 28.4	−39.1 ± 27.5	0.882
MCID Δ 0–6 months^b^	273 (84.8%)	54 (85.7%)	219 (84.6%)	0.818	54 (83.1%)	219 (85.2%)	0.668
MCID Δ 0–12 months^b^	290 (88.7%)	59 (93.7%)	231 (87.5%)	0.166	58 (89.2%)	232 (88.5%)	0.877
MCID Δ 0–24 months^b^	297 (85.1%)	57 (85.1%)	240 (85.1%)	0.995	60 (84.5%)	237 (85.3%)	0.875
PASS 6 months^b^	199 (61.8%)	34 (54.0%)	165 (63.7%)	0.154	34 (52.3%)	165 (64.2%)	0.078
PASS 12 months^b^	227 (69.4%)	38 (60.3%)	189 (71.6%)	0.081	37 (56.9%)	190 (72.5%)	**0.015**
PASS 24 months^b^	239 (68.5%)	39 (58.2%)	200 (70.9%)	**0.044**	43 (60.6%)	196 (70.5%)	0.108

*Note*: Twenty‐nine patients missed outcomes at 6 months, 24 patients missed outcomes at 12 months and nine patients missed outcomes at both 6 and 12 months. All patients had outcomes preoperatively and at 24 months. Bold values indicate statistically significant.

Abbreviations: n, number of patients; NRS‐Pain, Numeric Rating Scale for pain; MCID, minimal clinical important difference; PASS, patient acceptable symptom state.

^a^ Reported in mean ± standard deviation.

^b^ Reported in number (percentage).

Aseptic revision surgery was performed in five patients (1.4%). Revision rates were comparable for anxiety and depression patients, compared with nonanxiety and nondepression patients (Table 1).

## DISCUSSION

Main findings were that anxiety and depression patients had worse subjective function preoperatively and at all postoperative follow‐up moments, compared to the nonanxiety and nondepression groups. Furthermore, anxiety and depression patients had more pain preoperatively compared to their counters, and depression patients also reported more pain at all follow‐up moments. Finally, fewer patients with anxiety or depression reached the PASS for subjective function and pain. To our knowledge, this is the largest prospective cohort study with the longest follow‐up time assessing the influence of preoperative anxiety and depression, prior to TKA‐surgery, on pre‐ and postoperative subjective function, pain and revision rates.

Most prior studies focused on postoperative differences in subjective function and pain for anxiety and depression patients, rather than preoperative differences [16, 23, 34, 38, 41, 45]. However, some studies have reported on preoperative differences and had similar findings as the present study [4, 45, 47]. Vajapey et al. systematically reviewed that preoperative depression resulted in persisting joint pain and worse subjective function preoperatively [45]. Bierke and Petersen concluded similarly for anxiety patients that preoperative NRS‐Pain scores and subjective function were significantly worse [4]. Lastly, Wood et al. concluded that both anxious and depressed patients have significantly inferior subjective function and pain preoperatively [47].

Multiple prior studies obtained similar postoperative differences in subjective function and/or pain for anxiety and depression patients compared to nonanxiety and/or nondepression patients, as the present study [16, 23, 33, 38, 41, 47]. Subsequently, these studies found that anxiety and depression patients had worse pain and subjective function at the end of follow‐up, often at maximum 12 months. The study of Bierke and Petersen had similar results for preoperative anxiety [4]. Contrastingly, some prior studies did not find inferior postoperative subjective function in the depression and anxiety groups [20, 34]. However, one of these studies also included patients receiving nonprimary TKA surgery [20], which may have influenced their conclusions, since patient expectation will be different for patients receiving nonprimary TKA as opposed to primary TKA patients, while patient expectation is of considerably important influence on patient‐reported outcomes after total joint arthroplasty [17, 37, 42, 43].

In this study, a discernible pattern emerged wherein patients tend to achieve the MCID more frequently than the PASS. Notably, for patients not exhibiting anxiety, the PASS for KOOS‐PS is achieved more frequently than the MCID. However, this trend did not apply to nondepressive patients nor to OKS and NRS‐Pain outcomes for both nonanxious and nondepressive patients. Hence, it appeared that overall, the PASS is achieved less frequently and less ‘readily’ than the MCID. This could be explained by the fact that the MCID denotes a ‘threshold improvement’, while the PASS indicates a threshold at which the patient considers themselves ‘satisfied’, implying a higher level of contentment. Stated differently, the MCID pertains to ‘feeling better’, whereas the PASS relates to ‘feeling well’, which can vary significantly [11]. Therefore, it is suspected that generally patients find it easier to achieve the MCID than the PASS. A recent study by Bisson et al. on a different orthopaedic topic also found that significantly more patients achieved the MCID for KOOS than the PASS [5], possibly confirming this theory.

The current study showed an aseptic revision rate of 1.4% with no differences between anxiety and nonanxiety patients and depression and nondepression patients (*p* > 0.96). However, due to the low number of revisions, this study is likely to be underpowered for answering this research question. Moreover, it has been suggested by Mørup‐Petersen et al. that a difference in revision rates can unlikely be attributed to a difference in primary patient selection solely [31]. Prior studies rarely investigated differences in revision rates for anxiety or depression patients. This was, however, conducted by Sorel et al. and they concluded similarly that both anxiety and depression were not associated with revision surgery. However, it should be noted that they assessed anxiety and depression through EQ‐5D components instead of specific anxiety and depression PROMs, possibly underestimating the number of anxiety and/or depression patients [39].

In this study, 19% of patients had anxiety and 20% depression. One prior study had comparable anxiety and depression rates of, respectively, 20% and 23% [12]. And one study had considerably higher anxiety rates of 35%, but comparable depression rates of 22% [20]. Lower anxiety rates in our study are likely explained by cultural differences. Only Wood et al. had lower anxiety and depression rates of 15% and 11%, respectively; however, in their study, borderline anxiety and depression scores (scores 8–11) were categorized as ‘normal’, in contrast with the current study. According to the existing literature, when dichotomizing HADS scores, borderline scores should be categorized as ‘abnormal’ scores as well [6, 50].

LOS was significantly longer for anxiety and depression patients, which has also been reported by previous studies, accompanying higher medical costs [45], due to high daily cost of inpatient admission and increased risk of adverse events [18]. Moreover, more pain and poorer subjective function will result in prolonged use of analgesic medication [45, 47]. Although the most important reason for improving preoperative mental health remains the well‐being of patients, the consequences for healthcare costs must not be underestimated. Especially considering the globally increasing healthcare costs [19, 36, 46]. Various prior studies have already investigated different preoperative interventions. Some studies confirm this importance and suggest delaying the TKA procedure and adding psychological support prior to surgery [4, 40]. Other studies reject this importance and argue that TKA should not be postponed based on preoperative anxiety or depression [20, 28]. Based on the present results, we believe that preoperatively adding psychological therapy could indeed improve mental health and therefore improve preoperative and postoperative subjective function and pain.

The current study had several limitations. First, anxiety and depression were assessed only once preoperatively, and not postoperatively at any follow‐up moment. High HADS scores may have been a temporary phenomenon and not a true approximation of actual anxiety or depression. Moreover, preoperative anxiety or depression was only determined based on PROMs, possibly forging the results. Instead, proper psychiatric examination could have been performed preoperatively. However, possibly this would not have influenced the results drastically, as the rough distinction in patients with and without anxious or depressive features can very well be made with the HADS questionnaire [30, 49]. Additionally, strictly speaking, it cannot be said with certainty whether patients did not undergo therapy for anxiety or depression, as we did not explicitly ask this during this study. Furthermore, this was a single‐centre study and consequently, this may limit the generalizability of our findings. However, given that the distribution of patient demographics was similar to those reported in register‐based studies [13], we assumed that the present cohort was representative. Lastly, a survival analysis was not performed due to limited number of revision surgeries in the study population.

## CONCLUSION

Preoperative psychological factors, anxiety and depression, negatively influence preoperative and postoperative subjective function and pain up to 2‐year follow‐up in patients undergoing TKA, implicating the relevance of engaging psychological factors in decision‐making for surgical treatment and possible psychological intervention in the case of anxiety or depression preoperatively. Future research should focus on objectively, opposed to subjectively, assessing the impact of anxiety and depression on TKA outcomes and explore the potential benefits of preoperative psychological interventions through controlled trials.

## AUTHOR CONTRIBUTIONS

Margot B. Aalders designed the research question, performed analysis and interpretation of data, drafting and revising the manuscript and the final approval. Jelle P. van der List designed the research question, critically revised the manuscript and performed final approval. Lucien C.M. Keijser designed the research question, critically revised the manuscript and performed final approval. Joyce L. Benner designed the research question, performed acquisition, analysis and interpretation of data, drafting and revising the manuscript and the final approval.

## CONFLICT OF INTEREST STATEMENT

The authors declare no conflict of interest.

## ETHICS STATEMENT

Ethical approval for this study was obtained from the institutional review board (L019‐075) and written informed consents were obtained from all patients. The current study has been performed in accordance with the ethical standards in the 1964 Declaration of Helsinki. The study has been carried out in accordance with relevant regulations of the US Health Insurance Portability and Accountability Act (HIPAA).

## Data Availability

The data sets generated and analysed during the current study are not publicly available due to privacy and confidentiality agreements but are available from the corresponding author upon reasonable request. To ensure data privacy and ethical considerations, researchers requesting access to the data must submit a methodologically sound proposal and agree to use the data only for the intended research purpose. Access to the data will be granted to qualified researchers subject to a data use agreement. For data access requests, please contact Margot B. Aalders.
